# Large-scale climatic anomalies affect marine predator foraging behaviour and demography

**DOI:** 10.1038/ncomms9220

**Published:** 2015-10-27

**Authors:** Charles A. Bost, Cedric Cotté, Pascal Terray, Christophe Barbraud, Cécile Bon, Karine Delord, Olivier Gimenez, Yves Handrich, Yasuhiko Naito, Christophe Guinet, Henri Weimerskirch

**Affiliations:** 1 Centre d'Etudes Biologiques de Chizé, CEBC- UMR 7372 CNRS, Villiers en Bois 79360, France; 2 Institut Pluridisciplinaire Hubert Curien, Université de Strasbourg, UMR 7178 CNRS, 23 rue Becquerel 67087 Strasbourg, France; 3 Sorbonne Universités (UPMC, Univ Paris 06)-CNRS-IRD-MNHN, LOCEAN, 4 place Jussieu, Paris F-75005, France; 4 Centre d'Ecologie Fonctionnelle et Evolutive/CNRS, UMR 5175, 1919 Route de Mende, Montpellier 34293, France; 5 National Institute of Polar Research, 10-3 Midori-cho, Tachikawa, Tokyo 190-8518, Japan

## Abstract

Determining the links between the behavioural and population responses of wild species to environmental variations is critical for understanding the impact of climate variability on ecosystems. Using long-term data sets, we show how large-scale climatic anomalies in the Southern Hemisphere affect the foraging behaviour and population dynamics of a key marine predator, the king penguin. When large-scale subtropical dipole events occur simultaneously in both subtropical Southern Indian and Atlantic Oceans, they generate tropical anomalies that shift the foraging zone southward. Consequently the distances that penguins foraged from the colony and their feeding depths increased and the population size decreased. This represents an example of a robust and fast impact of large-scale climatic anomalies affecting a marine predator through changes in its at-sea behaviour and demography, despite lack of information on prey availability. Our results highlight a possible behavioural mechanism through which climate variability may affect population processes.

## Abstract (web summary)

Understanding how organisms respond to short-term climate variations will help predict the impact of future global change. Here, Bost *et al*. show that large-scale climatic anomalies coincide with changes in the foraging behaviour and populations dynamics of king penguins in the Southern hemisphere.

## Graphical abstract

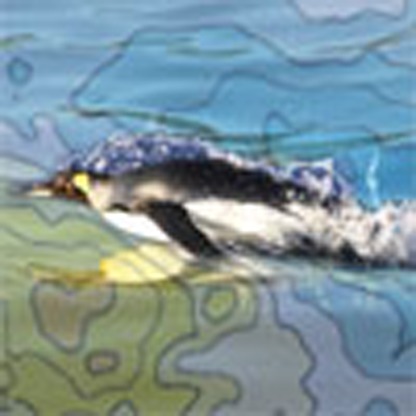

Long-term changes in population dynamics concurrent with climatic variability have been reported in an increasing number of wildlife species worldwide^1^,^2^,^3^,^4^,^5^. However, the primary causes and underlying behavioural mechanisms generating such population changes are rarely identified unequivocally.

In marine ecosystems, climatic anomalies such as the El Niño Southern Oscillation (ENSO) may drastically alter food webs^6^. Understanding how the organisms respond to such climatic variability is essential to assess the potential impact of global warming on the future of marine ecosystems. Long-term data sets on the dynamics of food webs are thus of key importance but few are available because of logistic difficulties and high costs.

The climate variability at subtropical and subantarctic latitudes in the South Indian and Atlantic Oceans is mainly controlled by subtropical dipole events^7^ partly linked to both ENSO and the Southern Annular Mode (SAM)^8^,^9^. These events are characterized by a tilted sea surface temperature (SST) anomaly dipole pattern oriented in the northeast–southwest direction in each basin, and are the leading mode of SST variability in both the southern subtropical Indian and Atlantic Oceans. How these subtropical large-scale climatic anomalies affect the ecosystems of the remote Southern Ocean is not known. In particular, the Southern Ocean holds considerable biomass of marine organisms including myctophid, one of the most important mesopelagic fish stocks in the world, which is yet poorly understood^10^. Several top predators rely on these mesopelagic fishes, especially in the Antarctic polar front (PF)^11^, a major boundary corresponding to the northern limit of Antarctic waters^12^. At the PF, the intense physical/biological interactions induce phytoplankton growth with subsequent increases in zooplankton biomass and concentrations of resources such as myctophid fish^13^.

Here we show how large-scale climatic anomalies in the Southern Hemisphere affect the foraging behaviour and population dynamics of a key predator of the Southern Ocean food webs, the king penguin (*Aptenodytes patagonicus).* First, we found that the occurrence of strong dipole events in the subtropical South Atlantic and Indian Oceans drives, at a very large scale, the temperature anomalies further south in the Southern Indian Ocean up to the polar frontal zone, which is the penguins' preferred foraging area^11^. Secondly, we studied the inter-year changes in penguins distribution and foraging activities in relation to the PF position by analysing a unique satellite tracking and diving database from tagged king penguins (1992–2010, 16 years of data). By linking these changes in penguins' at-sea activities together with related demographic data sets we show how this large-scale climatic variability immediately impacts the foraging activity and ultimately the demography of these predators.

## Results

### At-sea foraging activities of penguins

**Figure 1:**
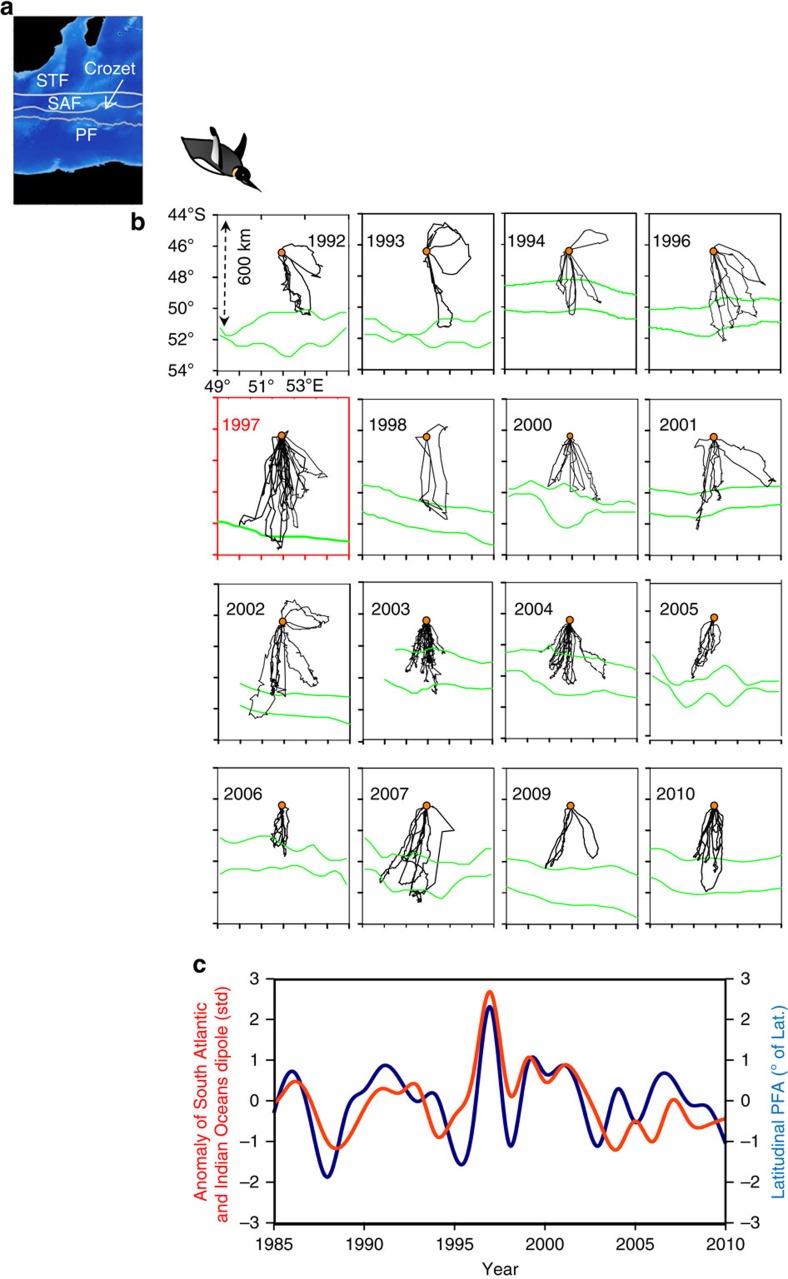
Foraging distribution of penguins and their relationship with large-scale climatic anomalies. (**a**) Map of the Crozet sector, South Indian ocean. The white lines show the main frontal structures. (**b**) Satellite-tracks of at-sea king penguins from the Crozet Islands monitored over a 16-year period (1992–2010). The tracks are shown with the corresponding locations of the Antarctic PF (green lines; upper line: 5 °C sea surface isotherm; bottom: 4 °C sea surface isotherm). The orange dots show the position of the study colony (Baie du Marin, Possession Island, Crozet). The red box displays the exceptional situation during the summer 1997 with the extension of the penguins' foraging ranges in relation to the southward shift of the PF. (**c**) Time series of latitudinal PF location anomaly and of the leading mode from the PCA of SST anomalies over a combined South Atlantic-Indian domain (10°–50°S, 50°W–150°E). See Fig. 3 for the associated SST spatial pattern. This leading PCA mode (standardized time series, red curve) of February–March SST anomalies described 27% of the SST variability over the combined domain during the 1979–2011 period and will be referred to as the South Atlantic and Indian Oceans dipole (SAIOD) time series. The anomaly of the PF zonal position (PFA, positive=south, negative=north, in degree, blue curve) concerns the latitude estimated for the sector between 50/54°E. SAF: Sub-Antarctic front; STF: subtropical front; PF: polar front.

The at-sea movements during the summer (from mid-December to mid-March) of foraging king penguins that breed on the Crozet archipelago, Southern Indian Ocean (Fig. 1a), were monitored annually from 1992 to 2010. The Crozet archipelago hosts the largest king penguins population (612 × 10^3^–736 × 10^3^ pairs) of the Southern Ocean^14^ (37%) (Supplementary Fig.1) which have an estimated myctophid consumption of 8 × 10^5^ tons/year^15^. When leaving the colony to forage, nearly all breeding individuals (93% of tracks) head south to forage (Fig. 1b). Foraging trips exhibits two distinct phases: a long distance commuting trips (300–500 km) to reach the northern boundary of the PF (Fig. 1b) and the time spent foraging at the PF^11^. To accurately quantify the penguins foraging success (number of prey catching attempts), we instrumented individuals with miniaturized data loggers allowing detection of feeding events (*n*=4 birds, Table 1) or predicting foraging success from diving behaviour (*n*=7 birds, Tables 2 and 3). Foraging success per day increased when the birds reached the PF. The catching success per foraging dive was variable but twice as high during the time spent at the PF than during the time spent commuting (see Methods, Tables 1 and 3, *P*<0.01).

### Consequences of the polar front variability

**Figure 2:**
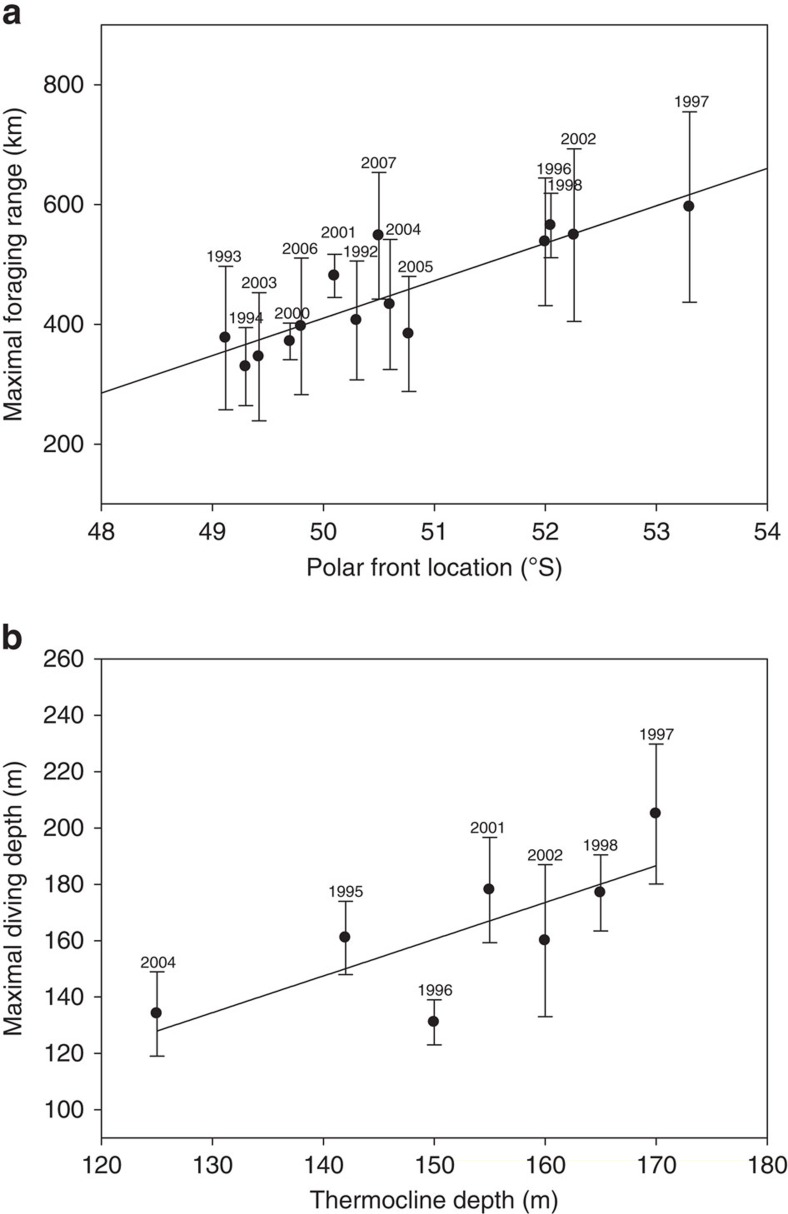
Relationships between penguins foraging behaviour and conditions at the Polar Front in summer, South Indian Ocean (Crozet sector). Error bars are s.e.m. and the solid black line are regression slopes based on the data. (**a**) Mean maximal foraging range of penguins as a function of inter-annual variations in the PF location (1992–2007, *n*=14 years). (**b**) Mean maximal foraging depth as a function of inter-annual variations in the thermocline depth in the polar frontal zone (1995–2004, *n*=7 years).

Between 1992 and 2010, SST anomalies affected the latitudinal position of the PF (Fig. 1). During the same period, penguin foraging ranges and the PF position exhibited extensive and concurrent variability (Fig. 1b, Fig. 2a; linear model, *F*_1,13_=38.3, *r*^2^=0.76, *n*=14, *P*<0.001). Warm anomalies considerably increased the distance covered by penguins to reach the PF (Fig. 2a). According to the link between SST anomalies and the PF location, an increase of 1 °C shifted its location southward by 130 km and the king penguin foraging range was similarly extended (data from the 1992–2010 period of penguins tracking). The travelling time from the colony to the PF was positively related to the PF location (linear model, *r*^2^=0.67, *n*=110, *P*<0.01). Penguins were more affected in terms of foraging distance when attending small chicks during late summer (linear model, *r*^2^=0.56, *n*=52, *P*<0.05) compared with the incubation period occurring earlier in the summer period (linear model, *r*^2^=0.30, *n*=58, *P*<0.05). The time to reach the PF according to its location (linear model, *F*_1,80_=30.6, *P*<0.01, Supplementary Fig. 2a,b) was not affected either by breeding stages or sexes (*P*>0.05). On the other hand, the time spent foraging at the PF according to its location (*F*_1,80_=23.2, *P*<0.01, Supplementary Fig. 2c,d) differed between breeding stages (*t*=−5.61, *P*<0.01) but not between sexes (*P*<0.05, Supplementary Fig. 2c,d).

In warmer years, the penguins not only went further but also they dived deeper (Fig. 2b). The SST changes in the oceanic area used by penguins induced concurrent changes in the depth of the thermocline (that is, the maximum of the vertical temperature gradient linked to the stratification of the water column^12^). The thermocline depth and topography are known to play a major role in fish aggregation^16^,^17^. Thermocline changes affected king penguin diving behaviour, which usually targets the depth of the thermocline^18^. Thus penguins dived deeper with increasing depth of the thermocline for a given year (Fig. 2b; linear model, *r*^2^=0.58, *n*=7 years, *P*<0.05).

### Relationships with large-scale climatic anomalies

**Figure 3:**
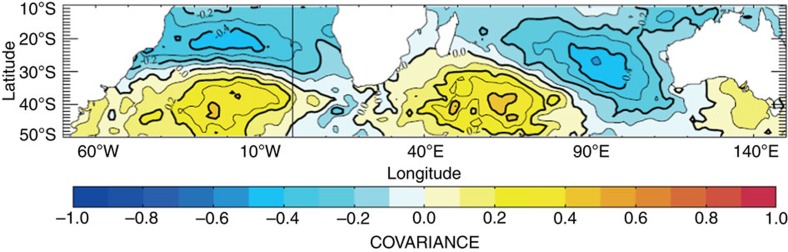
Spatial pattern associated with the Subtropical Atlantic and Indian Oceans dipole (SAIOD) time series (that is, the leading mode of the principal component analysis (PCA) of February–March SST anomaly fields over the combined South Atlantic–Indian domain).

During the study, the PF position was linked to large-scale SST fluctuations in the subtropical South Indian and Atlantic Oceans, as illustrated by the concurrent variability of the PF position and the leading mode of the principal component analysis (PCA) of the SST fields during austral summer (Fig. 1c). The maxima of this leading PCA mode indicate the occurrence of ‘positive' Subtropical Atlantic and Indian Oceans dipole (SAIOD) events (Fig. 1b) with positive SST anomalies in the southwest of the two basins (see Fig. 3).The latitudinal location of the PF was significantly associated with the occurrence of these positive SAIOD events during the summer (Fig. 1c; *r*^2^=0.63, *n*=26, *P*<0.001).

### Effects of the 1997 extra-tropical warm anomaly

An extreme positive SAIOD event occurred in 1997 with an abnormal large-scale warming of the southwest Indian Ocean, centred in the Crozet basin (Figs 1c, 3). As a direct consequence, an unprecedented southward shift of the PF occurred during the same year (Figs 1c, 2a).

**Figure 4:**
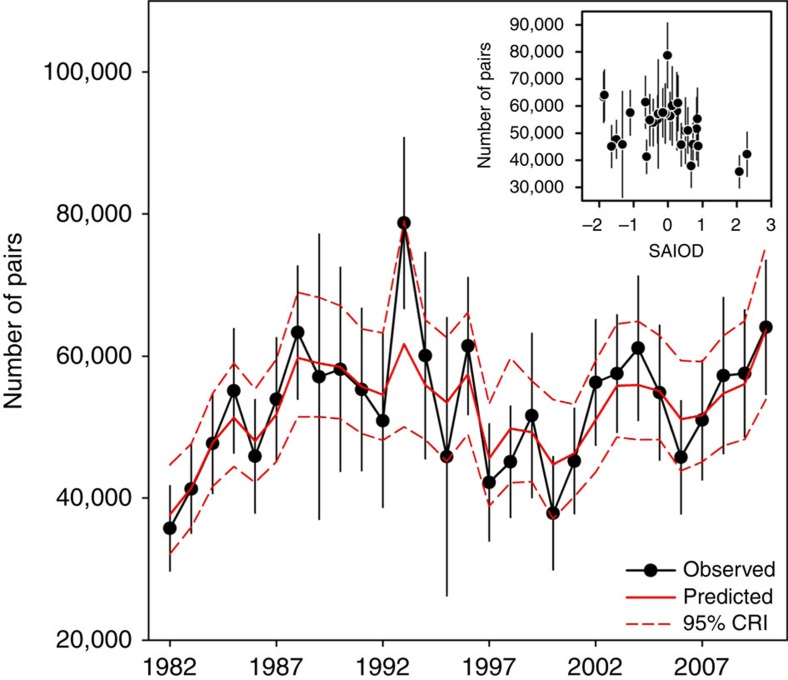
Observed and modelled changes in the king penguin breeding population at Possession Island (Crozet) from 1982 to 2010. The modelled changes were obtained from a stochastic Gompertz model with density dependence and an effect of the Subtropical Atlantic and Indian Oceans dipole (SAIOD) time series. The inset shows the observed breeding population size as a function of SAIOD. Error bars are s.e.m.

During the very strong positive SAIOD event of 1997, the mean foraging distances performed by the penguins rearing a chick (596±159 km) during summer (February–March) were the greatest reported over the whole study period, being doubled compared with the usual years (Fig. 1b). As the maximal foraging range corresponds to a high feeding activity of penguins^11^, the favourable foraging areas considerably shifted southwards as indicated by the tracking data. Diving behaviour was also affected in relation to such abnormal warming of the southwest Indian Ocean. During the same period *in situ* measurements from instrumented penguins travelling towards the PF indeed indicated a thermocline depth of ≈170 m (Fig. 2b), that is, 30 m deeper than during normal years^18^. As a consequence, the mean diving depth used by the penguins in the polar frontal zone was increased by 30.7% (Fig. 2b). Synchronously with these very unfavourable environmental conditions, the penguin breeding population experienced a 34% decline (Fig. 4) and recovered to their pre-event abundance only in 2002. This time period also corresponded to relatively low annual probabilities of adult survival^19^. Results from stochastic Gompertz population models (see Methods) indicate evidence of density dependence in the population, and suggest negative effects of SST anomalies, distance to the PF and SAIOD occurrences on breeding population size (Table 4). The decrease for the observed counts between 1993 and 1995 (and the increase between 1992 and 1993) partly reflects counting error which was probably higher in these years. The decrease between 2005 and 2007 can be explained by the low population size (and thus low chick productivity) in 5–6 years earlier (2000–2002) corresponding to the average age at first breeding of king penguins.

**Figure 5:**
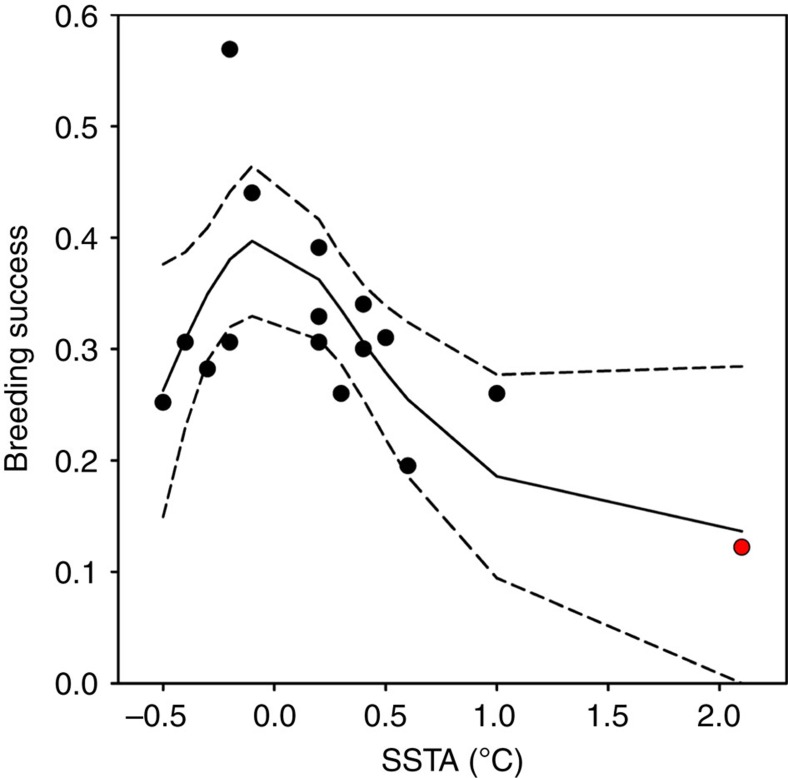
Fitted GAM results showing the relationship between the king penguin breeding success data and sea surface temperature anomalies (SSTA) in their main foraging area at the PF for the period 1987–2010. Dotted lines indicate 95% confidence intervals. The red dot indicate the year 1997.

There was also a negative effect of warm sea surface temperature anomalies (SSTA) on breeding success (Table 5), although the relationship was nonlinear, suggesting optimal SSTA in the main foraging area (Fig. 5). In particular the breeding success observed in 1997 was the lowest recorded over the whole study period. This strongly suggests that the increased foraging effort due to a southward shift of the PF has negatively affected some demographic parameters (recruitment and adult survival) of this long-lived species^19^, and therefore the population size. Thus, both physical and biological compartments of the southwest Indian Ocean responded immediately to this climate forcing.

## Discussion

Our results show that distinct large-scale climatic anomalies in the subtropical Indian and Atlantic Oceans had an immediate and major effect on the foraging habitat, diving behaviour, breeding success and population dynamics of a major top-predator of the Southern Ocean, probably through decreases in prey availability at lower trophic levels^20^. We provide evidence that the occurrence of strong dipole events in the subtropical South Atlantic and Indian Oceans drives, at a very large scale, the temperature anomalies further south in the polar frontal zone. These anomalies in turn affect king penguins in terms of at-sea distribution, foraging behaviour, breeding success and ultimately population dynamics.

Subtropical dipole events are the most important mode of SST variability in both the southern subtropical Indian and Atlantic Oceans, accounting 27% of the SST variability in these areas. Such modes of SST variability are controlled by the pulsations and shifts of the subtropical anticyclones and play even an active role at a global scale since they provide a remote control over ENSO variability in the equatorial Pacific^9^,^21^. SAIOD occurrence during the summer was significantly associated with the location of the PF. Importantly, these anomalous SST patterns are strongly phase-locked to the austral summer season, which is the breeding period where top predators are most dependent on the PF^11^. During this period, seabirds have to regularly return to the colonies to alternate with their incubating partner or for provisioning of the chick. Non-flying, swimming predators such as penguins are highly sensitive to environmental changes especially during the breeding period because of their low travelling speed, particularly during the breeding period characterized by limited foraging ranges^22^,^23^.

Climatic anomalies in the Southern Ocean were found to impact top-predator populations^7^,^19^,^23^,^24^,^25^,^26^,^27^ which potentially integrate environmental changes at various temporal scales being close to the apex of food webs^26^. In the northern hemisphere, several correlative studies have also reported impacts of large scale climatic anomalies on foraging success or demography of marine predators in diverse productive ecosystems. In northern Norway, movements of different water masses influence the water temperature which controls plankton abundance which in turns drives the growth of puffins' main prey, that is, first-year herring^28^. Long-term time series of productivity for several seabird species breeding in the California Current System indicate that the perturbation in the availability of food may have a complex origin. Thus ENSO has an important effect but other global atmosphere–ocean phenomena can also affect the local food webs just as dramatically^29^. However none of these studies showed a link between large-scale climatic events, foraging behaviour of a marine predator and its consequences on breeding success and population dynamics. Our study documents how extra-tropical climate anomalies may immediately affect a marine predator far in the Southern Indian Ocean by driving, at a very large scale, the temperature anomalies in the main feeding zone. It also established a link between large-scale climate changes, at-sea foraging behaviour, breeding success and population dynamics over a long-term scale, although direct data on the prey availability are lacking

Future climatic scenarios indicate a warming of the surface waters that should lead to a progressive southward shift of the PF^30^,^31^ and to a deepening of the thermocline^32^, thus impacting myctophid distribution and potentially representing a serious threat for penguins and other diving predators of the Southern Ocean. This highlights the value of retrospective analyses on top-predator life histories combining foraging and demographic parameters through different climatic/habitat scenarios^20^ and their potential to make robust predictions of the effect of climate change on ecosystems.

## Methods

### Penguin satellite tracking

Between 1992 and 2010, 6–15 breeding penguins from the king penguin colony of the Baie du Marin (20,000 pairs^33^), Possession Island, were fitted with Argos PTT Satellite Transmitters each austral summer, totalling 124 useable tracks. Such sample size was adequate with respect to the purpose of the tracking study according to the previous studies performed at the same colony^11^,^18^.The capture, release and handling procedures received the approval of the ethics committee of the French Polar Institute (IPEV) and of the French Environment Ministry. The birds were gently handled and the devices fixed to the feathers of the back with a cyanolycrate adhesive securely fastened with cables-ties. The equipment procedure took 15 min. We instrumented randomly selected any penguin observed relieved on their egg or small chick by its partner of breeding duties within the ten first ranks of breeders in the colony where birds can be accessed without disturbance. Almost the same number of females and males were instrumented each year. The penguins were sexed when relieved by their partner by using the inter sexes differences in mates call. Logistic reasons restrained tracking to only incubating birds in 1992, 1993, 1996, 2000 and 2001. The transmission interval ranged between 45 and 60 s. The front of all transmitters was hydrodynamically shaped and a flexible antenna, spring mounted, was used to limit the drag effect^34^,^35^. The transmitters weighted 1.8% of the mean adult weight. A track corresponds to the at-sea movements of a penguin during one foraging trip off the colony. Only birds having successfully foraged (no colony desertion) were kept for the analysis as failed breeders greatly increase their foraging range and the duration of their travels. No significant differences in breeding failure were reported among control and instrumented birds (respectively, 0.36 and 0.15%, *n*=34 and 23, *χ*^2^_1_=3.45, *P*=0.06). To compare the penguin foraging range to the PF location over the whole time series, we randomly selected the same number of incubating and brooding birds (early chick rearing period).

### Penguin feeding activities

To obtain detailed insights into the feeding activity of penguins, we used two kinds of information: (i) data from fast-response temperature sensors surgically implanted in the oesophagus together with time–depth recorders (1996/97, *n*=4 penguins, Table 1); and (ii) results from a predictive model of feeding success during penguins dives obtained from an another instrumented group (2009/10, *n*=7 penguins, early chick rearing) (Table 2). This was realized by combining different diving behaviours in a statistical procedure from the data sets obtained on the penguins instrumented in (i).

Concerning (i) we attached the oesophagus sensors together with a time–depth–temperature recorder on seven penguins at their departure to the sea. Oesophagus temperature loggers were attached to birds through surgical implantation^36^. A total of four birds provided extensive feeding records ranging from 6 to 9 days at sea (Table 1). Oesophageal temperature and depth were recorded with a resolution of 0.01 °C and 0.1 m, respectively. The pressure sampling rate was 2 s in the depth range 0–200 m and 4 s in the range 200–400 m. Experiments on captive individuals showed that fast-response temperature sensors were sensitive enough to detect prey similar to the smallest fish (1.8 g) caught by the free-ranging penguins. Temperature drops were characterized as feeding or non-feeding events with regard to amplitude and duration of the change in temperature^36^. Drops were characterized as feeding events for a 0.06 °C s^−1^ rate of temperature decrease^36^. Data were analysed using Jensen System Software programs (Laboe, Germany) and custom-made Matlab programs.

Concerning (ii), a generalized linear model was built to relate the feeding events (response variable) provided by four birds instrumented during early chick rearing period as indicated in (i) to different diving variables (explanatory variables): maximal depth, number of wiggles (quick variations of depth in three successive points during which the vertical speed passes below 0 m s^−1^, number of steps (periods within a dive during which vertical speed decreases to 0.35 m s^−1^, vertical ascent and descent rates and time spent at the surface previously a dive. Wiggles have been identified as reliable indicators of the feeding success^37^,^38^,^39^,^40^.

The penguins ‘vertical swimming speed (1.3 m s^−1^ during the ascent and descent phase of the dive) prevents any underestimation of wiggles and steps occurrence^37^. The analysis was conducted on the R statistical environment^41^. The overdispersion was tested (function *dispersiontest* package ‘AER') and a negative binomial family was choose (function odTest, *P*<0.0001, package ‘pscl'). Models were thus built using the *glm.nb* function (package‘MASS'). The explanatory variables were centred and scaled to facilitate the convergence of models and to compare the weight of different variables between them^42^. All possible combinations of variables were performed and classified according to the Akaike Information Criterion^43^. The QAIC was preferred in order to deal with the overdispersion of response variable. The *n* first models with the summed Q.Akaike weight *w* (probability of a model to be the best ‘true' model) inferior of 0.95 were submitted to a model averaging procedure (function *model.avg* package ‘MuMIn'). The full model-averaged coefficients (with shrinkage) and their adjusted s.e.m. are presented in Table 2. Complete models were built (two scales: dive; and diving bout: sequence of foraging dives, brooding stage). The identity of birds was not included as random effect as their number was too low. Thus, a model per individual was built in order to ensure that relation between the response and explanatory variables were similar to the complete models. Once models fitted, their predictive performance was estimated using a cross-validation procedure. The quality of the predictive power was given by the C.index which measures the probability of concordance between predicted and observed data (C.index, package ‘hmisc'^44^) (Table 2). At the diving bout scale, the strongest predictors of feeding events were wiggles (Table 2). The concordance index between observed data and predicted prey captures during diving bouts was ‘excellent' (C.index 0.90).

This predictive models was used to predict prey captures during diving bouts of an another penguin study group (*n*=7, early chick rearing period, 2009/10) during their foraging trip to the PF (Table 3). These penguins were instrumented both with an Argos transmitter and a time–depth–temperature recorder before going to sea. The birds were recaptured after 9–23 days spent at sea and the instruments recovered.

### Depth–temperature profiles recorded from penguins

We used temperature data from Mk5 and Mk9 data loggers (Wildlife Computers, Redmond, USA) attached to penguins foraging at the PFZ. The birds were caught in the same colony than the other studied groups. The loggers weighted 0.3–0.6% of the mean adult weight, respectively. The number of birds equipped was 8, 2, 3, 5, 6, 8 and 4 for the years 1995, 1996, 1997, 1999, 2001, 2002 and 2004, respectively (*n*=36, 20 males, 16 females). When animals were at sea, depth was sampled every 2 or 5 s and temperature was sampled every 5, 10 or 20 s depending on the sensors used. Depth was recorded with a 2 m resolution. The resolution of the different temperature sensors varied from 0.1 to 0.3 °C. The time constants (time required for the sensor to register 66.3% of the change in temperature) were found to be 6 s for Mk5 (refs. 45) and 1 s for Mk9 data loggers used. For Mk5 and MK9, 95% of the total temperature change was attained in less than 20 s. Thermocline depth corresponded to the modal depth reached by king penguins during foraging dives from spring to summer^18^). The time spent at the bottom of the dive (from 1.0 to 1.3 mn) allowed accurate estimations of the thermocline profile calculated^45^.

### Population parameters

The breeding population at Possession Island was inferred from the counts of breeding pairs of the four largest colonies on the island. These four colonies represented ≈80–90% of the total population breeding on the island and the abundance of breeding pairs varied in parallel at all four colonies^33^. The average distance between colonies was 10.3±5.9 km (min: 1.8 km, max: 15.0 km). Breeding pairs were counted using oblique photographs taken from promontories overlooking colonies in January (corresponding to the peak of laying period), when incubating birds are clearly distinct because they exhibit regular spacings between individuals^33^. Counts were not made every year at all colonies due to logistical reasons. The total number of counts included 65 colony.years (that is, there were 43.9% missing data). To obtain a complete time series of the number of breeding pairs from 1982 to 2011, we first combined the time series with missing observations from the four colonies, and performed a log-linear regression model with Poisson error terms using the program TRIM^46^. Missing counts were predicted from a model estimated on observed counts where year was entered as a discrete explanatory variable, taking into account overdispersion and serial correlation. We then used the stochastic Gompertz population model to analyse the effect of covariates on breeding population size while taking into account density dependence, since density dependence in this king penguin population was suggested by a previous study^33^, which suggested density dependence in adult fecundity and possibly on survival. By log transforming the population abundance in year *t* (*N*_*t*_) and putting *x*_*t*_=*ln*(*N*_*t*_), this model was defined through:


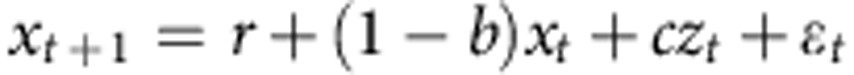


where 1−*b* is the lag 1 autocorrelation of the log transformed population abundance, *r* is the growth rate for *N*=1, *b* is a measure of the strength of density dependence, *z*_*t*_ is the value of a covariate in year *t*, and ɛ_*t*_ is normally distributed process error with mean zero and s.e.m. τ (refs 47, 48). When *b*=0 the process is density independent. The parameter *c* captures the effect of the covariate. When *c*>0 or *c*<0 the covariate has a positive or negative effect on population size, respectively.

Uncertainty in population abundance estimates was modelled with a log normal distribution so that the log transformed population abundance estimate in year *t* was given by *y*_*t*_=*x*_*t*_+η_*t*_, where *η*_*t*_ is sampling error with mean zero and s.e.m *σ*. Following^46^ models were fitted using a Bayesian approach implemented in R via JAGS^48^. We chose weakly informative normal priors for parameters *b* and *c*, and weakly informative uniform priors on the interval 0–3 for parameters σ and τ. As recommended by Lebreton and Gimenez^49^, we used an informative prior for *r* based on the demographic invariant approach^50^. This prior was a normal distribution with mean 0.10 and standard deviation 0.02. Inferences were drawn for posterior distributions based on 50,000 Markov Chain Monte Carlo simulations with two chains after a burn-in set of 10,000 updates. We assessed the convergence of the model fits by visually checking the trace of the posterior parameter estimates and computed Gelman and Rubin's R-hat convergence statistics.

In addition to population abundance we used annual breeding success data from earlier studies performed on the Baie du Marin colony of Possession Island 51,52,53,54,55, and unpublished data. Breeding success was obtained for 1987–1989, 1993, 1997–2004, 2006–2010) and estimated as the proportion of eggs laid by breeding pairs that fledged a chick. We investigated the relationships between breeding success and environmental covariates using non-parametric smoothing regression techniques^56^ (Table 4). Generalized additive models (GAM) were specified with a Gaussian family, used a penalized cubic regression spline, and the optimal amount of smoothing was estimated using cross-validation. The adjusted R-squared for the model was defined as the proportion of variance explained, where original variance and residual variance were both estimated using unbiased estimators. This quantity could be negative if the fitted model was worse than a one parameter constant model^56^.

To investigate the effects of environmental covariates on breeding success we performed generalized additive models.

### Environmental data

The time period considered for the climate analysis was 1979–2011. For the SST climatic analyses we use monthly mean data from the Hadley Centre Global Sea Ice and Sea Surface Temperature (HadISST1.1) data set^57^. PCA was used to extract the principal modes of variability of the SST fields^58^.

SST is a major variable determining king penguin foraging habitat^31^ and is highly correlated with other physical variables such as air temperature and sea ice extent. Surface isotherms are accurate indicators of the location of the PF, which is originally defined by the northern limit of Antarctic water. This subsurface water mass is measured by the northernmost extension of the 2 °C isotherm that corresponds to the 4–5 °C surface isotherms during summer^12^. We consider here the summer northern limit of the PF as the 5 °C surface isotherm^12^. Environmental covariates entered in the population model defined above were: (1) sea surface temperature anomalies (SSTA) south of Crozet in the polar frontal zone frequented by king penguins during summer (January-February)^18^; (2) distance to PF (Dist), calculated as the distance separating Possession Island to the 5 °C isotherm south of the island; (3) the southern Atlantic–Indian Oceans dipole (SAIOD) time series, which is defined as the first principal component of the PCA of SST anomaly fields over a combined South Atlantic–Indian domain (10°–50°S, 50°W–150°E) during February–March. The SSTA data were derived from satellite measurements AVHRR/MODIS E-W. The SSTA were estimated during February–March over the tracking period in a box south of Crozet corresponding to the area prospected by penguins over the whole period, i.e., between 47° and 53°S in latitude and between 49° and 55°E in longitude east and west of Crozet. Because SSTA, Dist and SAIOD were correlated, only one covariate at a time was entered in the stochastic Gompertz population model.

## 

## Additional information

**How to cite this article:** Bost, C. *et al*. Large-scale climatic anomalies affect marine predator foraging behaviour and demography. *Nat. Commun.* 6:8220 doi: 10.1038/ncomms9220 (2015).

## Supplementary Material

Supplementary Information Supplementary Figures 1-2

## Figures and Tables

**Table 1:** Feeding success of king penguins instrumented with a feeding recorder during foraging trips to polar front from Crozet Islands during early chick rearing period (1996/97 season).

**Bird**	**Feeding activity recorded (days)**	**Diving depth range (m)**	**Mean** ***N*** **prey ingested/day**			**Mean** ***N*** **prey/dive**			**Total number of feeding events**
			**polar front**	**Transit**	***P***	**polar front**	**Transit**	***P***	
B	6.3	56–199	392.0±186.6	86.6±68	<0.001	5.4±3.1	3.6±2.6	<0.0001	1,407
C	7.5	50–306	735.0±2.8	132.1±99.2	<0.001	5.3±3.6	1.8±2.1	<0.0001	2,342
G	6.1	56–199	169.5±50.2	25.5±19.7	<0.001	4.8±2.9	2.7±1.9	<0.0001	580
I	9.0	51–243	207.0±7.1	18.6±8.6	<0.001	4.7±2.6	1.8±1.2	<0.0001	544

*P* significance derived from Wilcoxon rank sum tests.

**Table 2:** Results of model averaging based on the best predictive models of prey capture established from diving parameters (at diving bout scales) of foraging penguins (early chick rearing period *n*=7, 2009/10).

**Final models—bout scale**	***w***	**Coef±s.e.m**	***P-value***
Intercept	**1**	**3.13±0.09**	0.00
Log (number of dives)	**1**	**1.45±0.18**	0.00
Log (Number of dives^2^)	**0.92**	**−0.25±0.12**	0.04
Maximal depth	0.50	−0.12**±**0.15	0.42
Number of wiggles	**0.97**	**0.32±0.11**	0.01
Ascent rate	0.20	−0.03**±**0.11	0.76
Descent rate	**1**	**0.47±0.12**	0.00
Number of step	0.45	−0.11**±**0.15	0.49
Surface duration	**0.87**	**−0.25±0.15**	0.09
C-index for fitted data	0.938	Excellent	
C-index for cross-correlation	0.923	Excellent	

The birds were instrumented with time–depth recorders and Argos transmitters. Coefficients (mean**±**adjusted s.e.m.) from the final generalized linear model selected after a cross-correlation procedure. *w*: probability for the variable to be present in the selected model. The values of the concordance index (C-index) are shown for model averaged on fitting data sets and after final cross-validation. Explanatory variables correspond to mean values except the number of dives. The best predictive variables are in bold. The number of diving bouts used to fit the model and to validate it were 36 and 38, respectively.

**Table 3:** Predicted feeding success of king penguins (*n*=7) both tagged with a time–depth–temperature recorder (sampling rate: 1 s) and an Argos transmitter during foraging trips to the polar front during the early chick rearing period (2009/10).

**Bird**	**Sex**	**Feeding activity recorded (days)**	**Mean number of prey ingested/day**			**Mean number of prey/dive**			**Total number of predicted feeding events**
			**polar front**	**Transit**	***P***	**polar front**	**Transit**	***P***	
B3	F	10	325±310 (6)	111±127 (4)	NS	3.1±2.0 (774)	1.6±1.2 (267)	<0.0001	2,404
W	F	9	161±51 (5)	65±41 (4)	<0.05	1.8±0.5 (490)	1.5±0.9 (168)	<0.05	1,308
N	M	13	366±318 (7)	134±128 (6)	NS	2.8±1.9 (1,038)	1.8±1.6 (498)	<0.0001	2,296
A5	M	21	24±190 (13)	131±145 (8)	NS	1.9±0.9 (1,496)	1.9±1.4 (611)	<0.0001	10,236
A7	M	22	318±236 (9)	139±94 (13)	NS	2.9±1.0 (996)	3.3±2.3 (889)	NS	7,550
A8	F	23	139±93 (13)	68±73 (10)	<0.05	1.8±0.8 (979)	1.4±0.7 (546)	<0.0001	7,905
O7	F	11	248±251c (4)	25±27(7)	<0.05	3.9±1.8 (276)	0.7±0.4 (179)	<0.0001	3,307

*P*-values derived from Mann and Whitney tests. Numbers in brackets are statistical effectives (days and dives) used for the tests.

**Table 4:** Stochastic Gompertz population models testing for the effects of density dependence, sea surface temperature anomalies, distance to the polar front and South Atlantic–Indian Oceans Dipole on the breeding population size of king penguins on Possession Island, 1982–2011.

**Model**	***r***	***b***	***c***	***Pr(c)<0***	***σ***	***τ***
DD+SSTA	0.101 (0.062; 0.141)	0.008 (0.002; 0.0133)	−0.020(−0.078; 0.044)	0.758	0.114(0.013; 0.185)	0.112(0.000; 0.206)
DD+Dist	0.100 (0.060; 0.139)	0.008 (0.002; 0.013)	−0.038(−0.107; 0.029)	0.870	0.106(0.007; 0.177)	0.120(0.024; 0.220)
DD+SAIOD	0.101 (0.063; 0.140)	0.008 (0.003; 0.014)	−0.067(−0.133; 0.005)	0.971	0.113(0.030; 0.187)	0.099(0.008; 0.192)

DD, density dependence; Dist, distance from the colony to the polar front; SAIOD, the south Atlantic–Indian Oceans dipole; SSTA, sea surface temperature anomalies. Values in parentheses indicate 95% highest probability density intervals for *r*, *b*, *c*, *σ* and *τ. Pr(c)<0* indicates the probability that the slope coefficient between population size and the covariate was negative.

**Table 5:** GAM results for the breeding success of king penguins from Possession Island as a function of SSTA, distance to the PF and SAIOD.

**Covariate**	***F*****-test**	***P*****-value**	**Adjusted** ***R*****^2^**
SSTA	4.572	0.023	0.417
Dist	1.711	0.217	0.124
SAIOD	2.730	0.102	0.187

Dist, distance from the colony to the polar front; GAM, generalized additive models; PF, polar front; SAIOD, the south Atlantic–Indian Oceans dipole; SSTA, sea surface temperature anomalies.
