# Supplementary material for: Large-scale climatic anomalies affect marine predator foraging behaviour and demography
Source: Nat Commun. 2015 Oct 27;6:8220. doi: 10.1038/ncomms9220 (PMC4639794; doi:10.1038/ncomms9220)
Supplement: Supplementary Information — Supplementary Figures 1-2 [file ncomms9220-s1.pdf]

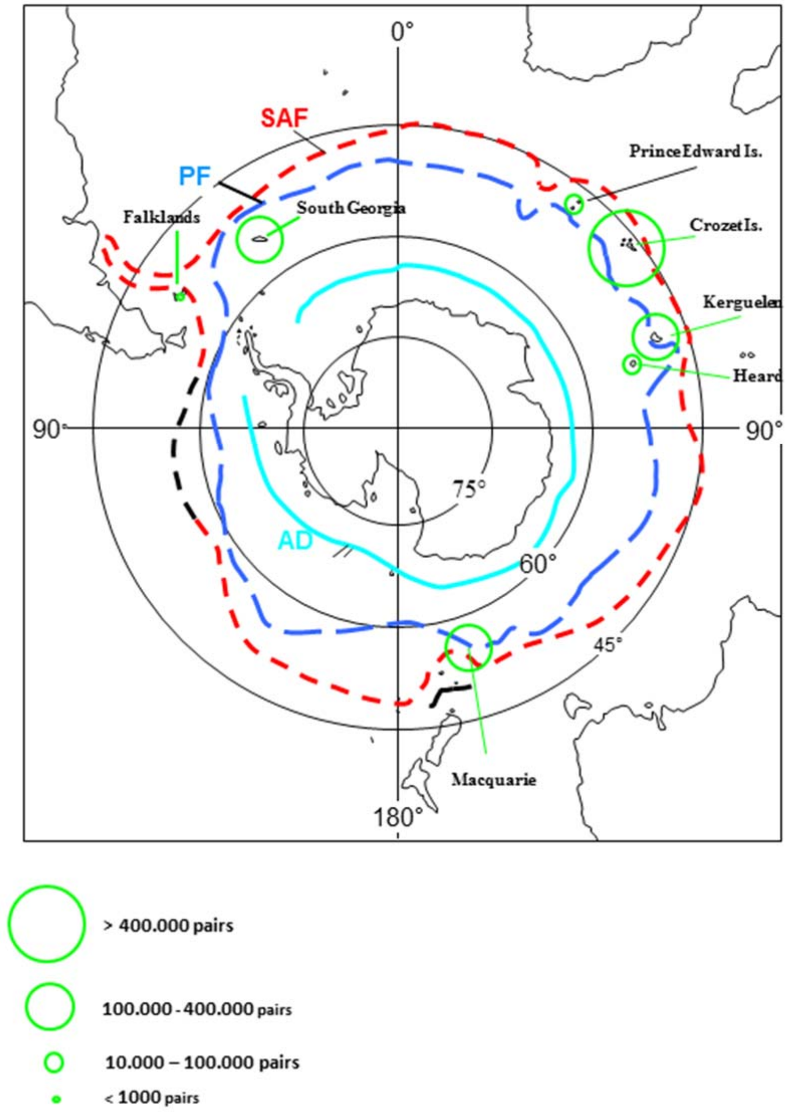

Supplementary Figure 1: Geographic distribution of King penguins in the Southern Ocean, including breeding localities. SAF: Sub-Antarctic Front; PF: Polar Front.

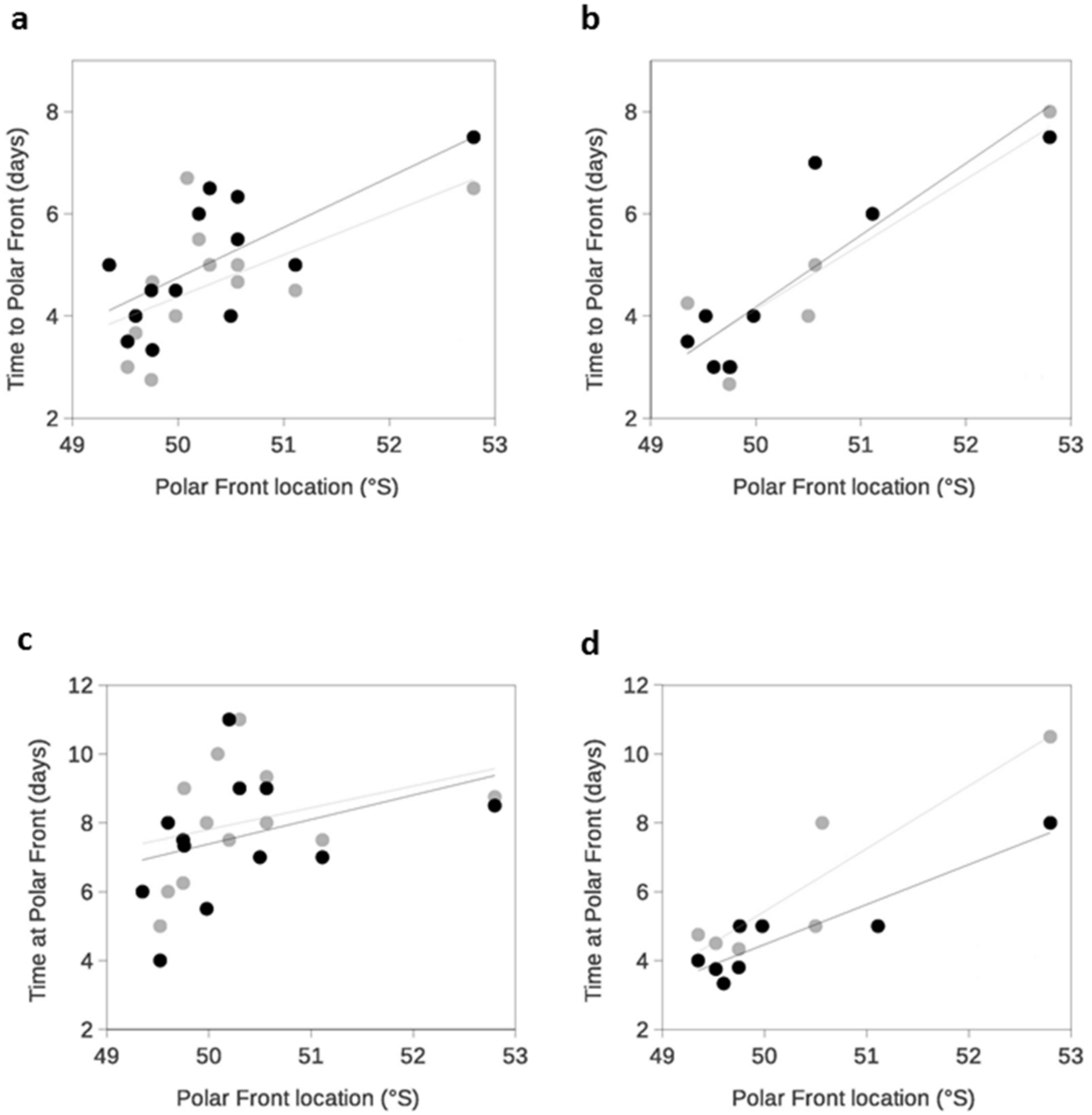

Supplementary Figure 2 : Linear regression of time spent travelling to the Polar Front; a: incubation (r^2=0,50 for females and r^2=0.37 for males); b: chick rearing (r^2=0,74 for females and r^2=0.81 for males) and time at Polar front; c: incubation (r^2=0,13 for females and r^2=0.11 for males); d: chick rearing(r^2=0,85 for females and r^2=0.85 for males)) by male and female king penguins according to the breeding stage (n=50 and n=34 during incubation and chick rearing respectively). The values of the mean time spent by males and females are represented by grey and black dots, respectively.
